# Evaluation of Polymorphisms in Toll-Like Receptor Genes as Biomarkers of the Response to Treatment of Erythema Nodosum Leprosum

**DOI:** 10.3389/fmed.2021.713143

**Published:** 2022-01-24

**Authors:** Miriãn Ferrão Maciel-Fiuza, Perpétua do Socorro Silva Costa, Thayne Woycinck Kowalski, Lavínia Schuler-Faccini, Renan Rangel Bonamigo, Rodrigo Vetoratto, Letícia Maria Eidt, Paulo Cezar de Moraes, Maria Irismar da Silva Silveira, Luis Marcelo Aranha Camargo, Sidia Maria Callegari-Jacques, Stela Maris de Jezus Castro, Fernanda Sales Luiz Vianna

**Affiliations:** ^1^Postgraduate Program in Genetics and Molecular Biology, Universidade Federal Do Rio Grande Do Sul, Porto Alegre, Brazil; ^2^Instituto Nacional de Genética Médica Populacional, Porto Alegre, Brazil; ^3^Genomics Medicine Laboratory, Center of Experimental Research, Hospital de Clínicas de Porto Alegre, Porto Alegre, Brazil; ^4^Laboratory of Immunobiology and Immunogenetics, Postgraduate Program in Genetics and Molecular Biology, Department of Genetics, Universidade Federal Do Rio Grande Do Sul, Porto Alegre, Brazil; ^5^Center of Social Sciences, Health and Technology, Universidade Federal Do Maranhão, Imperatriz, Brazil; ^6^Teratogen Information Service, Medical Genetics Service, Hospital de Clínicas de Porto Alegre, Porto Alegre, Brazil; ^7^Post-graduate Program in Pathology, Universidade Federal De Ciências Da Saúde de Porto Alegre, Porto Alegre, Brazil; ^8^Dermatology Service of Hospital de Clínicas de Porto Alegre, Porto Alegre, Brazil; ^9^Postgraduate Program in Medicine, Medical Sciences, Universidade Federal Do Rio Grande Do Sul, Porto Alegre, Brazil; ^10^Dermatology Service of Santa Casa Hospital of Porto Alegre, Porto Alegre, Brazil; ^11^Sanitary Dermatology Clinic, Secretaria De Saúde Do Estado Do Rio Grande Do Sul, Porto Alegre, Brazil; ^12^National Reference Center for Health Dermatology Dona Libania, Fortaleza, Brazil; ^13^Center for Research in Tropical Medicine, Porto Velho, Rondonia, Brazil; ^14^National Institute of Science and Technology-EpiAmo, Rondonia, Brazil; ^15^Department of Medicine, Centro Universitario São Lucas, Porto Velho, Rondônia, Brazil; ^16^National Institute of Science and Technology/CNPq-EpiAmo, Rondonia, Brazil; ^17^Department of Statistics, Universidade Federal Do Rio Grande Do Sul, Porto Alegre, Brazil; ^18^Postgraduate Program in Epidemiology, Universidade Federal Do Rio Grande Do Sul, Porto Alegre, Brazil

**Keywords:** leprosy, erythema nodosum leprosum (ENL), Toll-like receptor (TLR), thalidomide, prednisone

## Abstract

Erythema nodosum leprosum (ENL) is an inflammatory complication caused by a dysregulated immune response to *Mycobacterium leprae*. Some Toll-like receptors (TLRs) have been identified as capable of recognizing antigens from *M. leprae*, triggering a wide antimicrobial and inflammatory response. Genetic polymorphisms in these receptors could influence in the appearance of ENL as well as in its treatment. Thus, the objective of this work was to evaluate the association of genetic variants of *TLRs* genes with the response to treatment of ENL with thalidomide and prednisone. A total of 162 ENL patients were recruited from different regions of Brazil and clinical information was collected from their medical records. Genomic DNA was isolated from blood and saliva samples and genetic variants in *TLR1* (rs4833095), *TLR2* (rs3804099), *TLR4* (rs1927914), and *TLR6* (rs5743810) genes were genotyped by TaqMan real-time PCR system. In order to evaluate the variants' association with the dose of the medications used during the treatment, we applied the Generalized Estimating Equations (GEE) analysis. In the present sample, 123 (75.9%) patients were men and 86 (53.1%) were in treatment for leprosy during the ENL episode. We found an association between polymorphisms in *TLR1*/rs4833095, *TLR2*/rs3804099, *TLR4*/rs1927914, and *TLR6*/rs5783810 with the dose variation of thalidomide in a time-dependent manner, i.e., the association with the genetic variant and the dose of the drug was different depending on the moment of the treatment evaluated. In addition, we identified that the association of polymorphisms in *TLR1*/rs4833095, *TLR2*/rs3804099, and *TLR6*/rs5783810 with the dose variation of prednisone also were time-dependent. Despite these associations, in all the interactions found, the influence of genetic variants on dose variation was not clinically relevant for therapeutic changes. The results obtained in this study show that TLRs polymorphism might play a role in the response to ENL treatment, however, in this context, they could not be considered as useful biomarkers in the clinical setting due small differences in medication doses. A larger sample size with patients with a more genetic profile is fundamental in order to estimate the association of genetic variants with the treatment of ENL and their clinical significance.

## Introduction

Leprosy is a chronic infectious disease caused by *Mycobacterium leprae*. It especially injures the skin and peripheral nervous system, resulting in disabilities and characteristic deformities (1). Since leprosy has different clinical features, there are several systems of classification of the disease. The most used classification systems are Ridley-Jopling (based on clinical, pathological, bacilloscopic, and immunological criteria) and the World Health Organization (WHO) (a simplified operational classification to facilitate leprosy treatment) criteria. In the Ridley-Jopling system, patients are classified as indeterminate, tuberculoid, borderline, and lepromatous. In WHO system, patients are classified as paucibacillary (PB) and multibacillary (MB), according to clinical manifestation, being the latter more severe (2, 3). Around 50% of lepromatous leprosy (LL) and 5–10% of borderline-lepromatous (BL) patients develop inflammation conditions related do leprosy. One of them, called erythema nodosum leprosum (ENL), is characterized by painful subcutaneous erythematous nodules that can ulcerate and be associated to fever, general malaise and systemic inflammation (2, 4). ENL affects patients with poor cellular immune responses but with preserved humoral responses, and its immunopathological mechanism is not yet fully understood, although high levels of the pro-inflammatory cytokine tumoral necrosis factor-alpha (TNF-α) have been associated with the pathogenesis of the reaction (2, 5, 6). In order to manage the ENL symptoms, thalidomide and prednisone are drugs frequently used. Thalidomide has a rapid action in the symptoms control within 24–48 h, mainly due to the its anti-inflammatory action by inhibiting gene expression of TNF-α (2, 7, 8). In addition, the use of systemic corticosteroids may be necessary for patients with moderate, severe, and recurrent ENL (9, 10). They act in inflammation by suppressing or inhibiting the activation of the transcription nuclear factor kappa beta (NF-kβ), which regulates genes that encode various pro-inflammatory cytokines, including TNF-α (2, 4).

Interestingly, it has been postulated that the high levels of the pro-inflammatory cytokine observed in the ENL could be related to continuous recognition of *M. leprae* by Toll-like receptors (TLRs) and activation of an immune response (2, 11). After infection, *M. leprae* bacilli are initially recognized by several innate immune receptors, including TLRs. TLRs are membrane and endosomal receptors capable of interacting with several types of molecules, such as proteins and lipopolysaccharides (4, 12, 13). To date, ten members of the TLR family have been identified in humans, which are expressed in a variety of tissues and cell types throughout the body (14, 15). Each receptor recognizes different patterns and specific ligands of microbial components, having a pivotal role on the initiation of innate immune response (16, 17). Several studies indicate that recognition of mycobacteria by TLRs represents an essential step to produce an immune response, qualified to protect against infection (9, 18–21). TLRs 1, 2, 4, and 6 have been identified as capable of recognizing *M. leprae*, and, after interacting with the ligand, they trigger a wide antimicrobial and inflammatory response. The innate immune response influences the type of adaptive immune response induced by the expression of genes, which are mostly regulated by interaction of the TLR (22). Transcription factors activated by the TLR signaling pathways stimulate the expression of cytokines, such TNF-α and Interleukin 1 (IL-1), chemokines, and endothelial adhesion molecules (15, 23). Studies have revealed that thalidomide and its derivatives are effective inhibitors of TLR4-induced TNF-α production, and that the inhibitory effect of thalidomide on TLR-induced cytokines and type 1 IFN production may potentially contribute to the effectiveness of this drug in treatment of ENH (24–27). These findings indicate that the treatment alters the toll-like induced immune response. Thus, the recognition of *M. leprae* by the different TLRs determines the immune response profile of individuals and may be associated with the response to ENL treatment.

Polymorphisms in *TLRs* genes have been described and some of them might modify the levels of expression of the receptors, as well as their interaction with ligands or others receptors. Moreover, genetic variants in *TLR1, TLR2, TLR4*, and *TLR6* genes have been associated with autoimmune, infectious and inflammatory diseases, such as leprosy, pulmonary tuberculosis, atopic dermatitis, and systemic lupus erythematosus (6, 15, 22, 23, 28, 29). The single nucleotide polymorphism (SNP) rs4833095 of the *TLR1* gene results in an amino acid alteration from asparagine to serine. This alteration results in decreased expression of the TLR1 receptor in the immune system cells, altering the recognition of *M. leprae* (30, 31). Despite *TLR4* SNP, rs1927914, being located on the 5′ untranslated region (5′ UTR) of the gene, it can influence in regulatory processes, such as transcription factors' binding, altering the gene expression. It is believed that changes in the binding motifs of the transcription factors influence cellular responses related to TLRs (32, 33). SNPs in *TLR2* and *TLR6* were previously associated with leprosy or tuberculosis (14, 28, 34), but their specific roles are less understood. Due to the TLR relevance in inducing an immune response and previous association with infectious disease, in this study we investigated the association between thalidomide and prednisone doses with polymorphisms in *TLR* genes over time in the response to ENL treatment, in order to understand whether they could be useful as therapeutic molecular biomarkers.

## Materials and Methods

### Ethical Issues

All participants were informed about the research objectives and signed an informed consent form. This study was approved by the Ethics Committee of Hospital de Clínicas de Porto Alegre under CAAE 8989519.5.0000.5327.

### Sample

The sample consisted of 162 patients with ENL that were selected in seven Brazilian centers: at Hospital de Clínicas of Porto Alegre (*n* = 4), Dermatology Service of Santa Casa Hospital of Porto Alegre (*n* = 4) and at Sanitary Dermatology Clinic (*n* = 47), in Porto Alegre, South Brazil; at National Reference Center of Sanitary Dermatology Dona Libania in Fortaleza (*n* = 15), at Humanized Reference Center of Sanitary Dermatology (*n* = 13) in Imperatriz and Aquiles Lisboa Hospital in São Luís (*n* = 65) in Northeast Brazil, and at Dermatology Ambulatory of the University of São Paulo in Monte Negro (*n* = 14) in the north of Brazil. The inclusion criteria were ENL patients between 18 and 85 years-old, treated with thalidomide and/or prednisone. Patients using non-steroidal anti-inflammatory drugs were excluded.

### Clinical and Demographic Data Analysis

Up to ten visits previously registered in the patient's medical record were analyzed, with the collection of demographic data, including: sex, age, and place of origin, according to Brazilian political region; history of leprosy (moment of diagnosis and treatment); and history of ENL [diagnosis, types of treatment, adverse effects, history of relapse, and dose (mg) of medications used]. ENL was classified as acute, when a single episode lasting < 24 weeks; recurrent, if a second episode or following episode 28 days or more after the end of treatment; and chronic, when constant episodes were observed in more than 24 weeks (35).

### Genetic Analyses

Saliva and peripheral blood were used as biological material to DNA extraction. Genomic DNA was extracted from saliva samples with Oragene® kits (DNA Genotek), whilst peripheral blood DNA was obtained with the Flexigene® Blood Kit (QiagenTM) extraction kit, according to manufacturers' instructions.

Single nucleotide polymorphisms (SNPs) were selected based on minor allele frequency in each gene for Brazilian population and from the literature data regarding to the genetic association studies. The rs4833095 of *TLR1* (C_44103606_10), rs3804099 of *TLR2* (C_22274563_10), rs1927914 of *TLR4* (C_2704048_10), and rs5743810 of *TLR6* (C_1180648_20) SNPs were genotyped using the Real-Time PCR technique, with TaqMan assays (Applied Biosystems USA).

### Statistical Analyses

Clinical categorical variables were described in total number and percentage frequency. The medication doses were described using median and 25 and 75 percentiles. The Chi-Square Test was used to evaluate Hardy-Weinberg Equilibrium for all genetic polymorphisms and to compare the allelic frequencies of the polymorphisms, between the studied sample and the public database genetic variants in the Brazilian population (ABraOM).

To assess the association of variation in average doses of thalidomide and prednisone over time as a function of SNP, time and interaction between SNP and time, models of Generalized Estimating Equations (GEE) stratified by region (South and Northeast) were fitted. A total of 16 models were generated and the concomitant use of multidrug therapy (MDT) for treatment leprosy, use of other medications and other treatments for ENL were included as adjustment variables. The GEE method is a repeated measures analysis, focused on average changes in response over time and on the impact of covariates on these changes. The models were used with unstructured work correlation matrix, robust estimator of the covariance matrix and gamma distribution with logarithmic link function. The time variable is treated as quantitative in the models, so whenever that an interaction of the SNP with time is significant, the differences in the mean responses predicted by the model for the different categories of SNPs will be presented over time through a figure. In our analysis, if it is detected an interaction significant statistically between time and genetic variant analyzed, this result can be interpreted that average doses of the drug used in the ENL treatment are different depending on the moment of the treatment and the genotype of the individual. Therefore, every time this interaction is detected, we will describe it as a time-dependent association. SAS Studio was used to perform the Clopper-Pearson exact test. All other statistical analyzes were performed with SPSS version 18 (SPSS, www.spss.com, IBM, USA).

## Results

In this study, 162 patients with ENL were included, of which 123 (75.9%) were male and 144 (88.9%) had chronic ENL. About 75% (*n* = 121) of the patients presented LL, and 53.1% (*n* = 86) were using multidrug therapy during the treatment for ENL. Patients in the South (*n* = 45) and Northeast (*n* = 84) regions used other medications during treatment for ENL. The most common adverse effects were registered in the central nervous (14.8%), gastrointestinal (16.7%) and locomotor (24.2%) systems. The demographic and clinical characteristics of the sample are presented in Table 1. It was possible identify a large difference among the Brazilian geographical regions regarding to the clinical features of the patients and the treatment used.

**Table 1:** Clinical and demographic characteristics of ENL patients.

**Characteristic**	**South**	**North**	**Northeast**	***P*-value^€^**	**Total**
	**(*n* = 55) (%)**	**(*n* = 14) (%)**	**(n = 93) (%)**		**(*n* = 162) (%)**
Male	38 (69.1)	14 (100)	71 (76.3)	0.055	123 (75.9)
Concomitant Multidrug therapy for leprosy	39 (70.9)	13 (92.9)	34 (36.6)	<0.001	86 (53.1)
Use of other medications	45 (81.8)	0 (0)	84 (93.3)	<0.001	129 (79.6)
Prednisone dose [Median (P_25_-P_75_)]	40 (25–60)	50 (50–60)	40 (21.25–40)	$0.027^{\bar{\text{T}}}$	25.0 (20–40)
Thalidomide dose [Median (P_25_-P_75_)]	100 (100–200)	300 (200–300)	200 (100–200)	$0.005^{\bar{\text{T}}}$	100.0 (100–200)
Leprosy*					
Borderline-lepromatous (BL)	14 (25.9)	1 (7.1)	25 (26.9)	0.300	40 (21.5)
Lepromatous-leprosy (LL)	40 (74.1)	13 (92.9)	68 (73.1)		121 (74.7)
**ENL**					
Acute	5 (9.1)	0 (0)	5 (5.4)	0.005	10 (6.2)
Recurrent	7 (12.7)	0 (0)	1 (1.1)		8 (4.9)
Chronic	43 (78.2)	14 (100.0)	87 (93.5)		144 (88.9)
**Adverse effects**					
Central neurological^a^	9 (16.4)	0 (0)	15 (16.3)	0.292	24 (14.8)
Peripheral neurological^b^	1 (1.8)	3 (21.4)	16 (17.4)	0.012	20 (12.3)
Gastrointestinal^c^	7 (12.7)	0 (0)	20 (21.5)	0.075	27 (16.7)
Locomotor^d^	9 (3.6)	9 (14.5)	21 (27.3)	0.002	39 (24.2)
Ocular^e^	2 (3.6)	0 (0)	20 (21.7)	0.002	22 (13.6)
Edema	14 (25.5)	1 (7.1)	15 (16.3)	0.216	30 (18.5)
Cutaneous integument^f^	21 (38.2)	0 (0)	2 (2.2)	<0.001	23 (14.2)

* *n = 161/162*.

a *Dizziness and headache*.

b *Paresthesias and tremor*.

c *Diarrhea, vomiting and constipation*.

d *Myalgia and weakness*.

e *Decreased visual acuity and eye irritation*.

f *Pruritus, dry skin and hair loss*.

€ *Exact test using the monte carlo simulation method*.

*${}^{\bar{\text{T}}}$Kruskal-Wallis Test. ENL, erythema nodosum leprosum*.

Genotype distributions were tested for Hardy-Weinberg Equilibrium and the allelic and genotypic frequencies of the polymorphisms are shown in Table 2. *TLR4* (rs1927914) polymorphism was the only that was not in Hardy-Weinberg Equilibrium, representing an alteration in the allelic frequencies in the study population.

**Table 2:** Genotype and allele frequency of toll-like receptors.

**Gene**	Polymorphism^*^	**Genotype/allele**	**Number**	**Sample frequency (%)**	**ABraOM frequency (%)**	***P-*value^$\bar{\text{T}}$^**
*TLR1*	rs4833095 (T > C)	TT	40	24.7		
	Missense variant	TC	79	48.8		
	(Asn248Ser)	CC	43	26.5		
		T	159	49.07 (43.5–54.7)^†^	51	0.548
		C	165	50.93 (45.3–56.5)^†^	49	
*TLR2*	rs3804099 (T > C)	TT	51	31.5		
	Synonymous variant (Asn199 =)	TC	73	45.1		
		CC	38	23.5		
		T	175	54.01 (48.4–59.5)^†^	55	0.840
		C	149	45.99 (40.5–51.6)^†^	45	
*TLR4*	rs1927914 (G > A)	GG	40	24.7		
	2 KB upstream variant	GA	65	40.1		
		AA	57	35.2		
		G	145	44.75 (39.2–50.3)^†^	46	
		A	179	55.25 (49.6–60.7)^†^	54	0.841
*TLR6*	rs5743810 (A > G)	AA	11	6.8		
	Missense variant	AG	47	29.0		
	(Ser249Pro)	GG	104	64.2		
		A	69	20.66 (16.4–25.4)^†^	24	0.482
		G	265	79.34 (74.6–83.6)^†^	76	

* *TLR1, transcribed NM_003263; TLR2, transcribed NM_001318789; TLR4, transcribed NM_138554; TLR6, transcribed NM_006068*.

*${}^{\bar{\text{T}}}$Chi-square test between allele frequencies of the sample and the public database genetic variants in the Brazilian population (ABraOM)*.

† *Clopper-Pearson exact 95% confidence interval*.

Table 3 shows the average doses of thalidomide and prednisone, stratified by the patient's region of origin. To represent the evolution of the treatment, the averages of medication doses were described in three time periods, in the 1st consultation, between 75 and 105 days after the 1st consultation (about 3 months after the start of treatment), and between 165 and 195 days after the 1st consultation, ~6 months after starting treatment. In the North region, patients started medication with higher mean doses of thalidomide and prednisone than patients in the Northeast and South regions. For all regions, it was possible to identify a small reduction in the average dose of medications during the period evaluated. Despite this reduction, 41 patients were still using thalidomide, and 32 patients were still receiving prednisone 6 months after starting treatment.

**Table 3:** Average doses of medications by region and selected treatment periods.

		**Time intervals in relation to the start of treatment (1st consultation)**		
	**Region**	1st Consultation^*^	Between 75 and 105 days after the 1st consultation^*^	Between 165 and 195 days after the 1st consultation^*^
Thalidomide	South^†^	151.4 (64.6)	114.4 (63.1)	74.6 (33.8)
	Northeast^¥^	171.3 (73.5)	116.2 (53.8)	93.6 (65.5)
	North^$^	218.2 (75.1)	169.2 (63.0)	100.0 (0)
	Total	170.4 (73.0)	127.39 (59.5)	91.9 (62.8)
Prednisone	SouthK	43.7 (20.9)	21.2 (13.3)	26.6 (17.4)
	Northeast^ 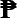 ^	36.4 (15.9)	25.1 (14.7)	20.2 (15.4)
	${\text{North}}^{\bar{\text{T}}}$	53.0 (11.6)	29.9 (15.5)	40.0 (0)
	Total	38.6 (17.4)	23.6 (14.2)	20.2 (11.4)

† *Number of thalidomide dose measurements per time interval in relation to the 1st consultation: 42, 44, and 15, respectively*.

¥ *Number of thalidomide dose measurements per time interval in relation to the 1st consultation: 92, 65, and 31, respectively*.

$ *Number of thalidomide dose measurements per time interval in relation to the 1st consultation: 11, 13, and 4, respectively*.

*
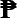
Number of prednisone dose measurements per time interval in relation to the 1st consultation: 34, 37, and 8, respectively*.

*KNumber of prednisone dose measurements per time interval in relation to the 1st consultation: 69, 56, and 31, respectively*.

*${}^{\bar{\text{T}}}$Number of prednisone dose measurements per time interval in relation to the 1st consultation: 10, 13, and 2, respectively*.

* *Means (Standard deviations)*.

**Figure 1:**
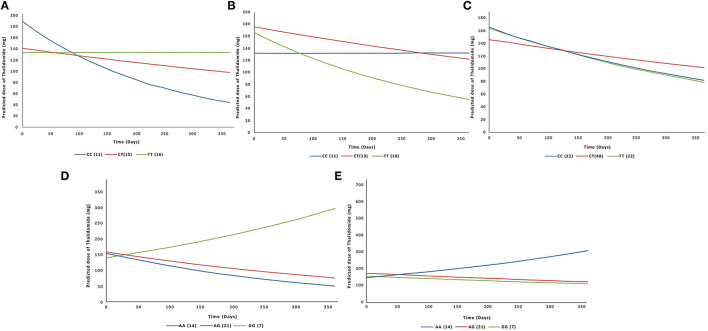
Lines charts of the thalidomide dose averages with Toll-like receptor genotypes discriminated by the times evaluated. **(A)** *TLR1*/ rs4833095 in the South region: blue line, CC genotype; red line, CT genotype; green line, TT genotype. **(B)** *TLR2*/rs3804099 in the South region: blue line, CC genotype; red line, CT genotype; green line, TT genotype. **(C)** *TLR2*/rs3804099 in the Northeast region: blue line, CC genotype; red line, CT genotype; green line, TT genotype. **(D)** *TLR4*/rs1927914 in the South region: blue line, AA genotype; red line, AG genotype; green line, GG genotype. **(E)** *TLR6*/ rs5783810 in the Northeast region: blue line, AA genotype; red line, AG genotype; green line, GG genotype.

GEE analyses were performed to assess the association of polymorphisms with the variation of the dose of thalidomide and prednisone over the time. Taking into account that triple interactions between the patient's region of origin, SNPs and time were observed, data were analyzed separately by geographical Brazilian region. Regarding treatment with thalidomide, we identified that, in the South region, the association of the genotypes of *TLR1*/rs4833095, *TLR2*/rs3804099, and *TLR4*/rs1927914 with the dose variation of the drug was time-dependent (interaction effect: *p* < 0.05; Table 4). For example, individuals with the CC genotype of *TLR1*/rs4833095 experienced a dose reduction throughout treatment (Figure 1A). In contrast, individuals with the AA genotype of *TLR4*/rs1927914 had increases in the medication dose during treatment (Figure 1D). In addition, we identified that, in the Northeast region, the association of the *TLR2*/rs3804099 (Figure 1C) and *TLR6*/rs5783810 (Figure 1E) genotypes with the variation of thalidomide dose also were time-dependent. For *TLR2*/rs3804099 we identified that individuals with CC and TT genotypes started treatment with a higher mean dose of thalidomide, but showed a greater reduction in mean dose over the treatment period when compared to CT heterozygotes. As for *TLR6*/rs5783810, individuals with AA genotype showed an increase in the average dose of thalidomide along the time of treatment evaluated, while individuals with the AG or GG genotype showed a reduction in the average dose. Regarding treatment with prednisone, we were able to identify that, in the South region; the association of the genotypes of *TLR1*/rs4833095, *TLR2*/rs3804099, and *TLR6*/rs5783810 with the dose variation of the drug was time-dependent. As for the Northeast region, only the association of the *TLR6*/rs5783810 genotypes with the dose variation was time-dependent (Table 4). Despite these interactions, we observed that, the difference in the average dose of both drugs, between the genetic variants evaluated, did not present impact on the ENL treatment. For example, the biggest difference in the average dose of thalidomide, between the genetic variants, was 52 mg among patients with genotype AA and GG of *TLR6*/rs5783810 in the South, representing only half of a tablet of the drug (100 mg) (Supplementary Table 1). We did not perform GEE analyses for the North region data due to the small sample size.

**Table 4:** *P*-values for the association of genotypes of *TLR* polymorphism, time and genotype^*^time interaction on dose of thalidomide or prednisone, as obtained in eight separate analyses for each drug and SNP using the Generalized Estimates Equation model (GEE).

				***P*** **-value for each association**		
**Drug**		**Gene**	**SNP**	**SNP** ${}^{\bar{\text{T}}}$	**Time** ^** 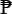 **^	SNP*Time ^€^
**South region**						
Thalidomide^a^	Model 1 (*n* = 37)	*TLR1*	rs4833095	0.054	0.004	0.011
	Model 2 (*n* = 37)	*TLR2*	rs3804099	<0.001	0.004	<0.001
	Model 3 (*n* = 37)	*TLR4*	rs1927914	0.630	0.162	0.011
	Model 4 (*n* = 37)	*TLR6*	rs5783810	0.214	0.002	0.948
Prednisone^b^	Model 5 (*n* = 37)	*TLR1*	rs4833095	0.049	0.001	<0.001
	Model 6 (*n* = 37)	*TLR2*	rs3804099	0.017	<0.001	0.013
	Model 7 (*n* = 37)	*TLR4*	rs1927914	0.891	0.096	0.093
	Model 8 (*n* = 37)	*TLR6*	rs5783810	0.009	<0.001	0.003
**Northeast region**						
Thalidomide^a^	Model 9 (*n* = 76)	*TLR1*	rs4833095	0.189	<0.001	0.766
	Model 10 (*n* = 76)	*TLR2*	rs3804099	0.129	<0.001	0.030
	Model 11 (*n* = 76)	*TLR4*	rs1927914	0.742	<0.001	0.250
	Model 12 (*n* = 76)	*TLR6*	rs5783810	0.303	0.136	<0.001
Prednisone^b^	Model 13 (*n* = 76)	*TLR1*	rs4833095	0.084	<0.001	0.273
	Model 14 (*n* = 76)	*TLR2*	rs3804099	0.483	<0.001	0.057
	Model 15 (*n* = 76)	*TLR4*	rs1927914	0.251	<0.001	0.470
	Model 16 (*n* = 76)	*TLR6*	rs5783810	<0.001	<0.001	<0.001

a Dependent variable: Dose of Thalidomide; Model: MDT, OM, Dose of Prednisone, Time, SNP, SNP^*^Time.

b Dependent variable: Dose of Prednisone; Model: MDT, OM, Dose of Thalidomide, Time, SNP, SNP^*^Time.

*SNP, single nucleotide polymorphism*.

*${}^{\bar{\text{T}}}$Main association of the SNP*.

*
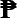
Main association of the Time*.

€ *Effect of the interaction between SNP and time*.

## Discussion

This study aimed to evaluate the association of genetic variants of TLRs genes with the response to the treatment of ENL with thalidomide and prednisone. We found that the association of *TLRs* genes polymorphisms with thalidomide and prednisone dose variation was time-dependent. These interactions between time and polymorphism mean that the association of the genetic variant with the dose of drug varies throughout the treatment, i.e., the association with the genetic variant is different depending on the moment of the treatment. Another interesting finding was the huge difference among Brazilian geographical regions regarding the treatment and clinical features of patients. This could be related to the different sample sizes, the heterogeneous genetic background of the Brazilian population and/or the treatment scheme used. Furthermore, during the statistical analyses, triple interactions between the patient's region of origin, SNPs, and time were observed. Thus, we decided to split our analysis by region. In spite of these regional differences, patients enrolled in this study were mainly male, diagnosed with lepromatous leprosy, chronic ENL, and using multidrug therapy and other medications during the treatment of ENL. The predominance of men is in accordance with the literature, which reports that proportionally more men are diagnosed with leprosy and ENL than women, being men more frequently affected by severe disabilities (36, 37). The greater number of patients with lepromatous leprosy, which is the form mainly associated with ENL, could indicate that the bacillary index is a risk factor for the development of the reaction as reported in other studies (36, 38, 39). In addition, the concomitant use of MDT in the present sample corroborate that the reaction manifests itself mainly in the first year of treatment of the disease (24, 40). Together, the findings support the hypothesis that the amount of soluble antigen during treatment, due to bacterial death, causes excessive activation of the immune system, inducing an inflammatory state, especially in those patients with high bacillary load at the beginning of therapy (2, 11).

Different constituents of *M. leprae* have been characterized as potent TLR ligands and stimulators of the immune system, such as the triacylated lipoproteins that have been indicated as activators of TLR2/TLR1 heterodimers and TLR2 homodimer (41). The *TLR1* gene encodes the receptor that recognizes *M. leprae* glycolipids and lipopolysaccharides and activates NF-kβ, which induces the expression of pro-inflammatory genes. The rs4833095 SNP involves a change from asparagine to serine at the position 248 of the protein, altering the expression of the TLR1 receptor in cells of the immune system (30, 31). Individuals with the T allele were shown to have decreased expression of TLR1 on the leukocyte surface. A defect in cell traffic is believed to affect the transport of the receptor to the cell membrane, altering its expression and recognition of *M. leprae*. Thus, the immune response generated becomes insufficient (31, 42). These data may indicate that individuals homozygous for the T allele of this SNP may show less signaling and inflammatory activity in response to *M. leprae*, resulting in the need for lower doses of anti-inflammatory drugs. Corroborating this hypothesis, in the present study we identified that, in the South region, despite having the dose reduced over the course of treatment, individuals with the CC genotype started treatment with an average higher dose of thalidomide (Figure 1A). This could indicate that individuals homozygous for the C allele of this SNP have greater inflammatory activity. However, we were unable to identify dose variation over time for individuals with CT and TT genotype. In addition, the dose reduction for individuals of the CC genotype was not significant for clinical management.

The SNP rs3804099 of *TLR2* is a synonymous polymorphism (43, 44). Synonymous variants can be functional by affecting the expression and activity of the receptor, by altering the transcription factor binding and/or the stability of the mRNA (43, 45). However, the exact mechanism related to the variant and *TLR2* activity remains unclear (45). Kang and Chae reported that the TT genotype of the rs3804099/*TLR2* polymorphism was associated with susceptibility to leprosy. The authors suggested that this variant (T) could affect the signaling function of the receptor, resulting in a deficient immune response (46, 47). In agreement, Santana et al. reported that the T allele of this SNP was positively associated with an increased risk of developing leprosy *per se* in a population in Northeast Brazil. In addition, in the population studied by Santana et al., carriers of the T allele were associated with higher serum levels of IL-17 and greater production of IL-6 (14). In this study, we identified that, in the South region, individuals with TT genotype started treatment with an average dose of thalidomide higher than those with CC genotype (Figure 1B). It may indicate that individuals homozygous for the T allele have an immunological profile with greater inflammatory activity, requiring higher doses of the drug. Moreover, they showed a dose reduction over the represented treatment period, while for individuals with CC genotype, we were unable to identify dose variation over the treatment period. Despite this, the reduction in the average dose, about 0.3% in the average dose/day, does not represent a clinical difference for medical management and the hypothesis about the role of T allele remains to be clarified.

The rs1927914 polymorphism of *TLR4* was not in Hardy-Weinberg Equilibrium. The Hardy-Weinberg principle is based on population genetics (48). Hardy-Weinberg deviation has been proposed as a measure of disease association when analyzing a disease group, since the individuals are a non-random selection of the population based on a phenotype of interest (49–52). In fact, leprosy has been associated with rs1927914 polymorphism (14), and that association could be explaining the Hardy-Weinberg imbalance found in our results. This finding could represent a bias; however, we did not remove it from of our analysis because the study sample was selected with leprosy patients, and therefore, it is possible that the risk allele is enriched in the entire sample. Moreover, we evaluated response to the treatment of ENL, hence the association between SNP and leprosy does not interfere in our analysis. The SNP rs1927914 of *TLR4* is located in the 5′-UTR. Thus, although the functional role of the SNP remains unknown, it is possible that it can influence gene expression, interfering with transcription factor binding and regulating the activity of the promoter (32, 53). Despite the classic TLR4 ligand being lipopolysaccharide (LPS), this receptor demonstrated the ability to recognize *M. leprae* and *M. tuberculosis* (9, 54). Santana et al. (14) observed significant differences in the levels of cytokines IL-17 and IL-1β in leprosy patients carrying the genotype AA, which produced more of both cytokines compared to individuals carrying the genotypes AG or GG together (14). In the present study, the results were different, because individuals with the GG genotype had an increase in the average dose of thalidomide over the course of treatment, when compared with individuals with the AA genotype. Clearly, other functional and molecular experiments with a genetic background different sample should be performed in order to understand the association between SNP rs1927914 and time in response to ENL treatment.

SNP rs5783810 is located in a coding region of *TLR6* and it promotes a change from serine to proline at the position 249 in the extracellular domain of the protein. This amino acid exchange results in the functional deterioration of TLR6 and predisposes individuals to dysregulation of the innate immune system (55, 56). Mattos et al. (20) suggested that TLR6-dependent signaling in interactions between *M. leprae* and Schwann cells plays a critical role favoring phagocytosis and signaling, to induce lipid droplet biogenesis in infected cells, thus providing mycobacterial survival and persistence of the microorganism in the nerve (20). The SNP rs5743810 of *TLR6* has been associated with susceptibility to tuberculosis among ethnic African, European or Hispanic groups (30). It has been proposed that this polymorphism influences the recognition of ligands and reduces cell signaling, with the A allele and the AG and AA genotypes, demonstrating to have a protective effect against the development of tuberculosis (30). In this study, individuals with AA genotype showed an increase in the average dose of thalidomide along the time of treatment, while individuals with the AG or GG genotype showed a reduction in the average dose over the course of treatment in the Northeast region. Lower cell signaling induced by the G allele of this SNP could imply less inflammatory response, resulting in the need to administer lower mean doses of the medication in individuals with the GG genotype. However, the group of individuals with the AA genotype is composed by a lower number of individuals, making the results interpretation difficult. Thus, further studies should be carried out to clarify the association between the SNP rs5743810 and the time in response to the treatment of ENL.

For prednisone treatment, we identified associations between polymorphisms in TLR genes and treatment time (Table 4). In the South region, we observed a negative association between the dose of the drug and the treatment time for the genotypes of *TLR1*/rs4833095 (CT and TT) and *TLR2*/rs3804099 (CC and CT). That is, there was a reduction in the average dose of the drug throughout the treatment. In addition, this association has also been identified in individuals with genotypes AA and AG of *TLR6*/rs5783810. In the Northeast, in all genotypes of *TLR6*/rs5783810, there was a negative association between dose and treatment time. However, in none of the interactions the mean dose variation was clinically significant, being with little or no impact on the clinical conduct of ENL.

Taking into account all results, we observed a reduction in the dose averages over the evaluated period. However, after around 6 months of treatment, 41 of the 134 patients who used thalidomide were still using the medication, whilst 32 of the 103 initial patients using prednisone remained receiving the drug. These data confirm that many individuals have chronic ENL, being treated with thalidomide and corticosteroids for prolonged periods of months. In addition, we showed that, in the three periods of time evaluated, patients of the North region received an average dose of both drugs higher than patients of the South region (Table 3). This finding could be related to the difference in sample size between the regions and/or the genetic heterogeneity of the Brazilian population (57, 58). However, it can also be related to the treatment regimen with thalidomide, since currently there is no standard dosage or indication for thalidomide dose reduction in the ENL in Brazil, and each center can adopt different and local protocols related to this medication (2, 39). Nevertheless, prolonged use of thalidomide and prednisone and/or high doses can cause serious adverse effects. Here, we grouped the adverse effects registered throughout of the treatment according to the symptoms. The most common were central nervous, gastrointestinal, and locomotor systems related. All of these effects could be associated with the use of thalidomide, since they are known causes of discontinuing the use of the drug (2, 59). However, we were unable to differentiate between adverse effects due to the use of thalidomide or prednisone, as well as those caused by leprosy. Intriguingly, we found no reports of sedation, the most common adverse effect associated with the use of thalidomide (60). This can be due to the lack of description of this effect in the patients' medical records, rather than the lack occurrence of the effect. Indeed, we noticed that the records of adverse effects were different when we stratifying the sample by region. Thus, the number of individuals with reports of specific adverse effects was significantly reduced in all the regions, making any causal inference about association between those effects and genetic factors likely spurious. For these reasons, we decided not to assess the association of *TLRs* with the occurrence of adverse effects related to the treatment of ENL with thalidomide and prednisone.

In this sense, there are other limitations in our study that must be taking into account in regard to the results interpretation here presented. Although we have had used a statistic model that controls confounding variables that could influence on the doses of the drugs, it is possible that other confounding factors may not have been considered in these models. One example is related to patient's origin. They were collected in different regions of the country and the heterogeneous genetic background and ancestry of the Brazilian population (57) may have been underestimated in the analysis and interpretation of the results. In addition, the limited sample size of this study can make it difficult to identify small effects of genetic variants in different outcomes for specific ancestries. Furthermore, this may also have influenced the large difference found between regions regarding to the average doses of thalidomide. Currently there is a wide therapeutic dose range of thalidomide for ENL in Brazil (100–400 mg/day) (61), and each center can adopt different protocols related to this medication and severity of disease. The analysis stratified by regions minimizes the potential biases of the different clinical settings. Finally, we conducted a retrospective study in which the patient's clinical data, therapeutic conduct and related adverse effects were obtained from medical records. Therefore, some important information could be missing in our analyses, because of the study design. The lack of standardization in the description of such information or the absence of reports cannot be excluded as well.

From the results of this study, we identified genetic variants in the TLR genes that may be associated with the response to treatment with thalidomide and prednisone. The association with the genotypes was dependent on the moment of treatment and patient's region of origin. However, the association of the SNPs on dose variation was not enough to suggest a change in the therapeutic conduct according to patient's genotype (Supplementary Table 1). ENL is a complex, chronic, and difficult to control condition, and all drugs used in its treatment require careful consideration (2). Thalidomide is an effective drug, but has use restrictions related to its teratogenicity and induced peripheral neuropathy (2, 62). Similarly, the prolonged use of corticoids causes several life-threatening adverse effects (63). Therefore, it is important to identify biomarkers that indicate response to treatment, in order to restrict the use of medications only to patients who will benefit the most of them. Here, we did not find biomarkers with clinical relevance to be used in the monitoring the treatment of ENL with thalidomide or prednisone. However, there are still many gaps to be filled in our knowledge of the interaction mechanism between the different TLRs and *M. leprae* and how these genes can interfere in the course and treatment of the disease. Nevertheless, despite TLR relevance in bacillus recognition, inducing an immune response and the association between genetic variants and ENL treatment showed here, genetic polymorphisms in TLRs may not be useful biomarkers in response to the treatment of ENL. Further studies analyzing the association of SNPs with the dose variation over time should be carried out to confirm this hypothesis, in larger samples with more homogeneous genetic features and assessment of ancestry.

## Data Availability Statement

Data cannot be shared publicly because of specific authorization was not asked to the patients. Requests to access the datasets should be directed to Hospital de Clinicas de Porto Alegre Institutional Ethics Committee, cep@hcpa.edu.br.

## Ethics Statement

The studies involving human participants were reviewed and approved by Ethics Committee of Hospital de Clínicas de Porto Alegre-HCPA. The patients/participants provided their written informed consent to participate in this study.

## Author Contributions

MM-F contributed to obtaining the specimens, designing and conducting the experiments, performing the statistical analyses, and writing the manuscript. PC contributed to devising the concept and designing the experiments. TK, LS-F, RB, RV, and LE contributed to designing the experiment and correcting the manuscript. PM, MS, and LC contributed to obtaining the specimens and correcting the manuscript. SC-J and SC contributed to designing the statistical analyses and writing the manuscript. FV contributed to devising the concept, designing the experiments, supervising the analyses, writing and correcting the manuscript. All authors discussed the results and contributed to scientifically to the manuscript.

## Funding

This work was supported by Instituto Nacional de Genética Médica Populacional (INAGEMP) (Grant Nos. CNPq 573993/2008-4 and FAPERGS 17/2551.0000521-0), Fundo de Incentivo à Pesquisa e Eventos (FIPE) of the Hospital de Clínicas de Porto Alegre (HCPA) (Grant No. 2019-0155), Coordenação Brasileira de Aperfeiçoamento de Pessoal de Nível Superior (CAPES), and Fundação de Amparo à Pesquisa do Rio Grande do Sul- FAPERGS (Grant No. 19/2551-0001787-1).

## Conflict of Interest

The authors declare that the research was conducted in the absence of any commercial or financial relationships that could be construed as a potential conflict of interest.

## Publisher's Note

All claims expressed in this article are solely those of the authors and do not necessarily represent those of their affiliated organizations, or those of the publisher, the editors and the reviewers. Any product that may be evaluated in this article, or claim that may be made by its manufacturer, is not guaranteed or endorsed by the publisher.
