# Supplementary material for: Evaluation of Polymorphisms in Toll-Like Receptor Genes as Biomarkers of the Response to Treatment of Erythema Nodosum Leprosum
Source: Front Med (Lausanne). 2022 Jan 24;8:713143. doi: 10.3389/fmed.2021.713143 (PMC8819000; doi:10.3389/fmed.2021.713143)
Supplement: Supplementary file 1 [file Table_1.DOCX]

Supplementary Material

# Supplementary Table 1: Average doses of thalidomide and prednisone stratified by region of patient´s origin and genotypes in Toll-like receptors genes.

|  |  | **Average thalidomide dose (mg)** | | **Average prednisone dose (mg)** | |
| --- | --- | --- | --- | --- | --- |
| **Gene/Polymorphism** | **Genotype** | **South (n)** | **Northeast (n)** | **South (n)** | **Northeast (n)** |
| *TLR1/*rs4833095 | CC | 173.0 (11) | 171.0 (28) | 21.0 (5) | 35.0 (22) |
|  | CT | 144.0 (15) | 168.0 (49) | 46.0 (15) | 38.0 (36) |
|  | TT | 144.0 (16) | 187.0 (15) | 49.0 (14) | 33. (11) |
| *TLR2/*rs3804099 | CC | 118.0 (11) | 193.0 (22) | 47.0 (9) | 38.0 (18) |
|  | CT | 169.0 (13) | 163.0 (48) | 45.0 (11) | 37.0 (36) |
|  | TT | 159.0 (18) | 173.0 (22) | 41.0 (14) | 33.0 (15) |
| *TLR4/*rs1927914 | GG | 157.0 (7) | 185.0 (26) | 33.0 (5) | 39.0 (24) |
|  | GA | 141.0 (21) | 172.0 (32) | 44.0 (20) | 38.0 (20) |
|  | AA | 154.0 (14) | 163.0 (34) | 50.0 (9) | 33.0 (25) |
| *TLR6/*rs5783810 | AA | 114.0 (7) | 200.0 (3) | 55.0 (2) | 60.0 (1) |
|  | AG | 140.0 (10) | 171.0 (26) | 44.0 (13) | 32.0 (19) |
|  | GG | 166.0 (25) | 171.0 (63) | 42.0 (19) | 37.0 (49) |
